# Effect of Gravitational Orientation on Bead Geometry and Position-Dependent Parameter Compensation in Multi-Pass Orbital Gas Tungsten Arc Welding

**DOI:** 10.3390/ma19194052

**Published:** 2026-09-22

**Authors:** Yuhyeong Jeong, Van Doi Truong, Jaehak Lee, Jonghun Yoon

**Affiliations:** 1 Department of Mechanical Engineering, Hanyang University, 55, Hanyangdaehak-ro, Sangnok-gu, Ansan-si 15588, Gyeonggi-do, Republic of Korea; 2 Autonomous Manufacturing & Process R&D Department, Korea Institute of Industrial Technology (KITECH), 143, Hanggaul-ro, Sangnok-gu, Ansan-si 15588, Gyeonggi-do, Republic of Korea; 3 Department of Mechanical Engineering, BK21 FOUR ERICA-ACE Center, Hanyang University, Ansan-si 15588, Gyeonggi-do, Republic of Korea

**Keywords:** orbital GTAW, gravitational orientation, bead geometry, welding position, position-dependent control

## Abstract

This study investigated the influence of circumferential welding position and process parameters on bead geometry during multi-pass orbital gas tungsten arc welding (GTAW) of SUS304 stainless-steel pipes. Three-dimensional surface profiles were measured using a laser line scanner at the top (12H, −45° to 45°) and bottom (6H, 135° to 225°) circumferential regions. The incremental bead height and width of Passes 1 and 2 were quantitatively evaluated after excluding the first and last 10% of the measured region to minimize edge effects. Under identical root-pass conditions, the average Pass 1 bead height at 6H was 9.6% greater than that at 12H, whereas the bead width was 3.3% smaller. After Pass 2, the height difference increased to 12.0%, while the width difference remained small at 2.4%. Primary current, wire feed speed, and travel speed exhibited position-dependent effects on bead formation. Primary current affected the balance between vertical buildup and lateral spreading, wire feed speed consistently influenced bead width, and travel speed strongly affected bead buildup through changes in heat input, filler deposition per unit length, and molten-pool residence time. The greater reinforcement at 6H was attributed to gravity-dependent molten-metal accumulation during successive deposition. Interpolation of the primary-current experiments predicted that approximately 173 A at 12H and 175.5 A at 6H would produce comparable Pass 2 geometries of approximately 1.62 mm in height and 6.57 mm in width. These results demonstrate that circumferential bead uniformity can be improved through position-dependent parameter adjustment rather than identical welding conditions around the pipe circumference.

## 1. Introduction

Gas tungsten arc welding (GTAW), also referred to as tungsten inert gas (TIG) welding, is widely used for joining stainless steels and other engineering alloys because of its stable arc characteristics, controllable heat input, and capability to produce high-quality welds [1,2,3]. These characteristics make GTAW particularly suitable for applications requiring high joint integrity and dimensional consistency, including high-quality pipe welding. However, the resulting weld geometry is strongly affected by process variables such as welding current, travel speed, pulse conditions, and filler metal supply, which govern heat input, molten-pool dimensions, penetration, and solidification behavior [4,5,6,7]. For pipe welding, orbital GTAW enables automated circumferential movement of the welding torch and provides improved process repeatability and consistency. Previous studies on orbital GTAW have demonstrated that welding current, travel speed, pulse parameters, welding position, and other process variables significantly affect bead geometry, penetration, and weld quality [1,2,3,4,5]. More recent investigations have confirmed these parameter sensitivities specifically for orbital TIG welding of 304 series stainless-steel pipes. Minh et al. investigated SUS304 orbital TIG welding and reported substantial effects of welding current and travel speed on heat distribution and weld geometry [1]. More recently, Tran et al. investigated filler-wire and current conditions in orbital TIG welding of 304 stainless-steel pipe and demonstrated that heat input and filler-wire conditions significantly influence the resulting weld characteristics [8]. These recent results further emphasize that parameter selection in orbital welding must consider the coupled effects of energy input and filler metal deposition rather than relying on a single fixed parameter. In particular, investigations of SUS304 and AISI 304L stainless-steel pipes have shown that welding current and travel speed strongly influence heat distribution and the resulting weld geometry [1,2]. Therefore, appropriate control of welding parameters is essential for achieving consistent bead formation during orbital welding.

A fundamental challenge in orbital welding is that the orientation of the molten pool relative to gravity continuously changes as the torch travels around the pipe circumference. Consequently, even when identical welding parameters are maintained, molten-pool behavior and the resulting bead geometry can vary with circumferential position [3,9,10,11,12]. Experimental and numerical investigations have demonstrated that gravitational orientation affects free-surface deformation, molten-metal redistribution, penetration, and final weld geometry [9,10,11,12]. Kang et al. demonstrated pronounced differences in weld-pool deformation and penetration under different gravitational orientations [9], while subsequent studies on 304 stainless steel confirmed that weld-pool shape and surface deformation depend on welding position, current, and heating duration [10,11]. Gravity-induced sagging and asymmetric bead formation have also been reported in horizontal narrow gap GTAW of stainless steels [12]. These findings indicate that maintaining identical process parameters throughout an orbital weld does not necessarily guarantee uniform bead geometry.

The positional dependence of bead geometry originates from the complex interaction of driving forces within the molten pool. Previous numerical and experimental studies have separately examined the principal physical mechanisms governing GTAW pool behavior. Marangoni convection, driven by surface-tension gradients, has been shown to strongly affect the direction and intensity of molten-metal circulation [13,14,15]. Electromagnetic force and buoyancy further modify the internal flow field and heat transport within the molten pool [16,17], while arc pressure and plasma shear stress influence free-surface deformation and lateral molten-metal redistribution [18,19,20,21]. These studies demonstrate that the relative contribution of each driving force depends on welding current, material properties, molten-pool dimensions, and gravitational orientation. Consequently, the final bead geometry results from the combined action of these physical mechanisms rather than from nominal heat input alone. Recent experimental studies further support the strong dependence of GTAW bead geometry on process conditions. Yildirim et al. demonstrated through response surface methodology that variations in electrode and arc-related parameters significantly alter bead width and penetration [22]. In pulsed GTAW-based deposition, recent investigations have likewise demonstrated that current-related parameters strongly influence bead width, bead height, and penetration, confirming that the final geometry reflects the combined thermal and fluid-flow response of the molten pool [23]. The influence of gravity is also coupled with the thermal state and lifetime of the molten pool. When the molten pool remains liquid for a longer period, gravity has more time to redistribute molten metal before solidification [9,10,11,12]. For example, Kang et al. reported greater free-surface deformation at lower scan velocities, demonstrating the interaction between the time available for molten-metal flow and gravitational orientation [9]. Welding position and process parameters should therefore not be treated as independent factors. Instead, changes in current, filler metal supply, or travel speed can produce different geometrical responses depending on the local orientation of the molten pool relative to gravity. Primary welding current is one of the principal parameters controlling arc energy, melting, and molten-pool development. Increasing the current generally increases the thermal and mechanical effects of the arc and can modify penetration, pool fluidity, and lateral bead spreading [1,2,6,13,14,15,16,17]. However, increasing current does not necessarily produce a proportional increase in bead height because increased energy can simultaneously promote melting, free-surface deformation, and lateral redistribution of molten metal [9,13,14,15,16,17,18,19]. Consequently, the geometrical response to current variation must be considered in conjunction with molten-pool dynamics and welding position. Filler metal supply is another important factor controlling bead formation. In filler-wire GTAW, wire feed conditions affect the quantity and transfer behavior of metal supplied to the molten pool and consequently influence deposition geometry [13,24,25,26]. Studies of hot wire GTAW have demonstrated a close relationship between metal transfer behavior and weld geometry [24], while wire-fed GTAW deposition studies have demonstrated the importance of wire feed conditions for dimensional control [25]. Depending on the available thermal energy, wetting behavior, and molten-pool flow, the supplied filler metal may contribute to either lateral spreading or vertical buildup.

Travel speed has a particularly complex influence because it simultaneously changes heat input per unit length, filler metal deposition per unit length, and molten-pool residence time [6,9]. Increasing travel speed generally reduces the thermal energy supplied per unit length and shortens the time available for molten-metal redistribution, whereas decreasing travel speed prolongs thermal exposure and molten-pool lifetime [6,9]. Travel speed can therefore affect not only overall bead dimensions but also the extent to which gravity influences molten-metal redistribution.

The combined influence of welding position and process parameters is particularly important in circumferential pipe welding. Figueirôa et al. investigated orbital TIG welding of steel pipes and demonstrated that welding position, current mode, and current magnitude influenced the geometrical characteristics of the deposited beads [3]. Together with other orbital welding studies [1,2,3,4,5], these findings suggest that applying a single parameter set throughout the circumference may not always produce uniform bead geometry and that local process conditions may need to be adjusted according to circumferential position. This issue becomes more important in multi-pass welding because the geometry produced by one pass forms the physical boundary condition for subsequent deposition. Recent multi-layer and multi-pass welding studies have increasingly emphasized this geometrical dependency between successive passes. Wang et al. incorporated the pre-weld geometric constraint profile together with welding parameters to predict the complete cross-sectional profile of subsequent weld beads, demonstrating the importance of the existing geometry as an input condition for subsequent deposition [27]. Similarly, recent data-driven research on multi-pass multi-layer welding has shown that considering the initial and previously formed geometry is important for accurately predicting the subsequent weld-bead profile [28]. These findings reinforce the need to evaluate bead geometry on a layer-by-layer basis rather than treating individual passes as geometrically independent events. Variations in bead height or width can modify the remaining groove geometry, local wetting conditions, and molten-metal redistribution during the next pass [12,13,14,15,16,17,18,19]. Consequently, a geometrical deviation generated during an early pass may persist or influence the formation of subsequent layers, potentially resulting in cumulative geometrical nonuniformity during multi-pass circumferential welding.

Three-dimensional surface measurement provides an effective method for quantitatively characterizing these spatial variations. Laser- and vision-based sensing techniques have been widely employed for weld-bead-profile measurement, defect detection, seam tracking, and geometric inspection [29,30,31]. More recently, advanced vision and laser line-scanning techniques have been developed for high-resolution and automated reconstruction of bead and deposited-track geometry. Recent vision-based research has emphasized the acquisition of molten-pool and weld-bead-profiles for automated welding monitoring [32], while laser line-scanner-based approaches have demonstrated high-resolution three-dimensional geometry monitoring and reconstruction of deposited surfaces [33]. These developments demonstrate the increasing importance of spatially resolved geometric sensing for quantitative process monitoring and provide further motivation for the laser line-scanner-based bead characterization adopted in the present study. Structured light measurement enables the weld-bead profile to be evaluated without physically sectioning the specimen, while laser-based vision systems can reconstruct geometrical features from three-dimensional weld profiles [31,32]. Such measurements are therefore well suited for quantitatively comparing spatial variations in bead height and width at different circumferential positions.

Although SUS304 is a well-established austenitic stainless steel whose general GTAW characteristics have been extensively investigated, an important issue remains in multi-pass orbital welding. Previous orbital welding studies have demonstrated that welding current, travel speed, pulse conditions, and welding position affect bead geometry, penetration, and weld quality [1,2,3,4,5]. However, comparatively limited attention has been given to the layer-by-layer quantitative evolution of bead geometry at different circumferential positions during multi-pass orbital GTAW. This issue is particularly important because the geometry produced by one pass forms the physical boundary condition for subsequent deposition, and variations in bead height or width can modify the remaining groove geometry, local wetting conditions, and molten-metal redistribution during the following pass [12,13,14,15,16,17,18,19,34,35]. Furthermore, previous optimization studies have primarily focused on identifying a common set of process parameters that improves bead geometry, penetration, or overall weld quality [1,2,3,4,5], whereas comparatively little attention has been directed toward position-dependent welding parameters specifically intended to compensate for gravity-induced geometrical differences.

Therefore, this study quantitatively investigates the effects of circumferential welding position and process parameters on bead formation during multi-pass orbital GTAW of SUS304 stainless-steel pipes. Three-dimensional surface profiles at the 12H and 6H positions were measured using a laser line scanner, and the incremental bead height and bead width were evaluated for Pass 1 and Pass 2. For Pass 2, primary current, wire feed speed, and travel speed were independently varied relative to a reference condition to determine their effects on bead geometry and their interaction with circumferential position. Finally, the experimentally observed parameter sensitivities were used to derive position-dependent welding conditions aimed at minimizing the differences in bead height and width between the 12H and 6H positions. Rather than assuming that uniform circumferential welding requires identical process parameters at every position, the present study investigates a geometry-oriented compensation strategy in which welding conditions are adjusted according to circumferential position to obtain comparable final bead geometry. This approach provides a basis for the future development of position adaptive control strategies for automated multi-pass orbital GTAW.

## 2. Experimental Procedure

### 2.1. Experimental Setup

Orbital gas tungsten arc welding (GTAW) experiments were performed using an automatic orbital welding system (Model 227, AMI, Pacoima, CA, USA). High-purity argon gas (99.9%) was employed as the shielding gas at a flow rate of 15 L/min. The filler wire was SUS308L with a diameter of 0.9 mm. SUS304 stainless-steel pipes with an outer diameter of 203.2 mm (8 inch) and a wall thickness of 8.2 mm were used as the base material. Austenitic stainless-steel SUS304 pipes with an 8-inch diameter were selected as the base material because they are widely used in industrial piping systems requiring high weld quality. The chemical composition of the SUS304 pipe used in the present study is summarized in Table 1. A V-groove joint with a groove angle of 60° was machined at the center of the pipe prior to welding to simulate a practical multi-pass pipe welding configuration.

To investigate the influence of welding position on bead geometry, welding was conducted under orbital conditions in which the torch continuously rotated around the circumference of the pipe. The present study focused on comparing the overhead position (12H) and the bottom position (6H), where the influence of gravity on molten-pool behavior is expected to be significantly different. The 12H region was defined as the circumferential range from −45° to 45°, with 0° corresponding to the top of the pipe, whereas the 6H region was defined as the range from 135° to 225°. The circumferential regions corresponding to the 12H and 6H positions used in the present analysis are illustrated in Figure 1. All welding experiments were performed using identical equipment and environmental conditions to minimize external variations, allowing the observed differences in bead geometry to be attributed primarily to the welding position. The orbital welding and line scanner configuration used in the experiments is shown in Figure 2.

### 2.2. Welding Conditions

To experimentally verify the influence of welding position, identical welding parameters were first applied to both the 12H and 6H positions. The reference welding condition was established as the baseline condition for stable bead formation. Starting from this reference condition, each welding parameter was independently increased and decreased while the remaining parameters were maintained as constant. During preliminary trials, the parameter was progressively varied until observable welding defects began to occur. Accordingly, the upper and lower parameter levels listed in Table 2 were selected as the limiting conditions immediately preceding the onset of observable welding defects. This procedure defined the parameter variation range used to evaluate the individual effects of primary current, wire feed speed, and travel speed on bead geometry while excluding conditions that produced apparent welding defects. The reference welding condition consisted of a primary current of 180 A, a background current of 105 A, a travel speed of 2.54 mm/s, a wire feed speed of 9.31 mm/s, and a welding time of 66 s.

After evaluating the bead geometry obtained under the reference condition, additional experiments were conducted by varying individual welding parameters while maintaining the remaining parameters constant as shown in Table 2. In Table 2, the bold value in each C1–C6 condition indicates the welding parameter intentionally varied relative to the reference condition, whereas the remaining welding parameters were maintained at their corresponding reference values. To minimize the influence of residual heat from the preceding welding operation, the specimen was allowed to cool to room temperature after each individual bead welding operation before the subsequent welding experiment was performed. Accordingly, each successive welding operation was initiated after cooling rather than being deposited continuously on a specimen maintained at an elevated inter-pass temperature. This procedure was applied consistently to all experimental cases to minimize thermal accumulation and to provide comparable initial thermal conditions for evaluating the effects of circumferential position and welding parameters on bead geometry. The primary current was adjusted to 170 A (C1) and 190 A (C2), whereas the wire feed speed was modified to 10.16 mm/s (C3) and 8.47 mm/s (C4). Furthermore, the travel speed was changed to 2.12 mm/s (C5) and 2.96 mm/s (C6). This one-factor-at-a-time approach was adopted as an initial sensitivity analysis to isolate the effect of each welding parameter on bead geometry at the two circumferential positions. The present experimental design was not intended to establish a comprehensive multi-parameter process model, and interactions among primary current, wire feed speed, and travel speed were therefore not independently quantified. A systematic design of experiments incorporating parameter interactions will be required in future work to establish a more comprehensive position-dependent process model. This approach enabled quantitative evaluation of the influence of each welding parameter on bead geometry and provided a basis for identifying parameter adjustments capable of minimizing positional differences in bead formation.

### 2.3. 3D Bead Measurement

The three-dimensional geometry of the welded beads was measured using a laser line scanner (LJ-X8080, KEYENCE Corp., Osaka, Japan) mounted directly on the orbital welding head. Because the scanner rotated together with the welding torch, the surface profile of the weld region could be acquired immediately after each welding pass without repositioning the specimen or changing the experimental configuration. The laser line scanner is a non-contact optical measurement system with a measurement range of 73 mm in the X direction and 20.5 mm in the Z direction. The profile sampling interval along the X direction was 12.5 μm, and the repeatability was 1 μm and 0.5 μm in the X and Z directions, respectively. The high spatial resolution of the system enabled detailed reconstruction of the groove and weld-bead surfaces and allowed small geometric differences caused by the circumferential welding position to be quantified.

For each experimental condition, the groove profile before welding (Pass 0) and the surface profiles after the first and second deposited passes (Pass 1 and Pass 2) were sequentially measured. One welding experiment was conducted for each experimental condition. During each experiment, the laser line scanner continuously acquired transverse profile data according to its measurement frequency as the scanner moved along the circumferential welding direction. Consequently, more than 600 spatially resolved profile datasets were obtained for each experimental condition. These profiles were used to quantitatively evaluate the spatial variation in bead height and width within the welded region. Therefore, although each welding condition was independently tested once, the mean values and standard deviations reported in the following sections were calculated from more than 600 spatial profile datasets for each experiment and represent within-weld spatial variability rather than variation among independent welding repetitions. Each dataset consisted of a series of transverse line profiles acquired along the circumferential welding direction. These profiles were reconstructed into three-dimensional surface data and subsequently used for quantitative evaluation of weld geometry.

The geometric features of each pass were first extracted independently from the actual measured profiles rather than from direct profile subtraction. Pass 0 was used to determine the initial groove geometry, whereas Pass 1 and Pass 2 were independently analyzed to identify the corresponding bead peak and toe locations. Based on these measured geometric features, the bead height of each deposited pass was subsequently calculated from the difference between the vertical distances of consecutive profiles. This approach preserves the actual geometric features of each measured profile while enabling the incremental vertical buildup produced by each welding pass to be quantified.

The profile analysis therefore determined five primary geometric quantities: (1) the Pass 0 center-to-outer distance, (2) the Pass 1 peak-to-outer distance, (3) the Pass 2 peak-to-outer distance, (4) the Pass 1 bead width, and (5) the Pass 2 bead width. Based on these quantities, the incremental bead heights of Pass 1 and Pass 2 were calculated from the corresponding vertical distance differences.

### 2.4. Data Processing and Geometric Parameter Extraction

To quantitatively evaluate the effect of circumferential welding position on bead formation, the acquired line scanner profiles were processed using a dedicated geometric analysis algorithm. The overall procedure consisted of profile preprocessing, outer surface reference estimation, groove region identification, bead peak detection, toe detection, local artifact correction, and extraction of the final geometric parameters.

Direct superposition of the Pass 0, Pass 1, and Pass 2 profiles was not used to determine the incremental bead dimensions. As illustrated in Figure 3, the measured profiles may exhibit changes in the absolute depth level and local surface geometry between successive passes. These differences can arise not only from deposited weld metal but also from welding-induced thermal deformation, dimensional changes in the pipe, and small variations in the measurement reference. Consequently, simply subtracting or directly comparing the superimposed profiles could introduce errors in the determination of the individual bead height and width. To minimize these effects, the geometrical features of each pass were identified independently from its measured profile. The outer surface reference, bead peak, and left and right toe positions were determined separately for each pass, and the incremental bead height and bead width were subsequently calculated from these geometrical features according to the definitions described in the following sections. This feature-based approach enables the geometry of each deposited pass to be evaluated without relying on direct point-to-point differences between successive profiles.

#### 2.4.1. Outer Reference Determination

For each transverse profile, the relatively flat base material regions located outside the weld groove were identified on both sides of the weld zone. A linear regression was applied independently to the left and right outer surface regions, and the resulting fitted lines were used as local outer surface references. For Pass 0, the center of the initial groove was identified, and the vertical distance between the groove center and the corresponding outer reference was calculated. This value was defined as the Pass 0 center-to-outer distance, $D_{0}$. The overall region is illustrated at Figure 4.

#### 2.4.2. Groove Region and Bead Peak Detection

Pass 1 and Pass 2 were processed independently using their actual measured profiles. The groove region was first identified as the continuous region located below the corresponding outer reference line. Within this region, the deposited weld bead was localized, and the highest valid point of the smoothed bead profile was defined as the bead peak.

This procedure avoids using a global maximum outside the weld region and prevents unrelated outer surface features from being incorrectly identified as the weld-bead peak. The vertical distances from the corresponding outer reference to the detected bead peaks were defined as the Pass 1 peak-to-outer distance, $D_{1}$, and the Pass 2 peak-to-outer distance, $D_{2}$, respectively.

The bead height of each deposited pass was calculated from the vertical distance difference between consecutive measured profiles. Because the groove and bead profiles were acquired using the same measurement coordinate system, the incremental vertical buildup produced by each welding pass could be determined from the corresponding center-to-outer and peak-to-outer distances. For clarity, $D_{0}$ represents the vertical distance from the Pass 0 groove center to the outer reference, while $D_{1}$ and $D_{2}$ represent the vertical distances from the Pass 1 and Pass 2 bead peaks to their corresponding outer references, respectively. For Pass 1, the bead height was defined as the difference between the Pass 0 center-to-outer distance and the Pass 1 peak-to-outer distance:

$$H_{1}=D_{1}-D_{0}$$

where $H_{1}$ is the Pass 1 bead height, $D_{0}$ is the Pass 0 center-to-outer distance, and $D_{1}$ is the Pass 1 peak-to-outer distance.

Similarly, the Pass 2 bead height was defined as the difference between the Pass 1 and Pass 2 peak-to-outer distances:

$$H_{2}=D_{2}-D_{1}$$

where $H_{2}$ is the Pass 2 bead height and $D_{2}$ is the Pass 2 peak-to-outer distance.

Accordingly, the Pass 1 and Pass 2 bead heights represent the incremental vertical buildup produced by each pass, rather than the absolute vertical position of the bead peak relative to the pipe outer surface. The bead peak and peak-to-outer distance definitions are illustrated in Figure 5. This definition enables the contribution of each deposited pass to the progressive filling of the groove to be quantitatively evaluated.

#### 2.4.3. Toe Detection and Bead Width Calculation

After the bead peak was identified, the left and right toe positions were determined by searching outward from the bead peak toward the adjacent groove slopes. The toe was defined as the nearest physically valid local minimum corresponding to the transition where the deposited weld bead meets the adjacent welding groove. The profile was searched outward from the bead top toward the groove on each side. When a distinct local minimum was present, this minimum was identified as the corresponding toe position. When the bead-to-groove transition formed a locally flat minimum region rather than a single distinct minimum, the point within the flat region closest to the bead top was selected as the toe. This criterion provides a consistent definition of the bead boundary for both distinct and locally flat bead-to-groove transitions.

The bead width was calculated as the horizontal distance between the detected left and right toe positions, as illustrated in Figure 6. The same procedure was independently applied to the Pass 1 and Pass 2 profiles, yielding the Pass 1 and Pass 2 bead widths, respectively.

## 3. Results and Discussion

### 3.1. Experimental Verification of Positional Effect

The effect of circumferential welding position on bead formation was first evaluated by comparing the surface appearance and three-dimensional bead profiles obtained at the 12H and 6H positions. Although the same welding procedure was applied around the circumference, noticeable differences in bead shape were observed between the upper and lower welding positions. In general, the bead formed at 12H exhibited a relatively flat and laterally distributed profile, whereas the bead at 6H tended to exhibit greater vertical buildup and a more concentrated bead shape.

This positional difference was already observable after the root pass. Because Pass 1 was deposited using the same welding conditions for all experimental cases, the variation observed in the root-pass geometry cannot be attributed to changes in welding parameters. Instead, it indicates that circumferential position itself affects molten-pool behavior and solidification. After excluding the first and last 10% of the measured region to minimize transient edge effects, the average Pass 1 bead height for the C1–C6 cases was 1.347 mm at 12H and 1.477 mm at 6H. Thus, the bead height at 6H was approximately 9.6% greater than that at 12H. In contrast, the corresponding average bead widths were 6.185 and 5.983 mm, respectively, indicating that the 6H bead was approximately 3.3% narrower. The positional dependence became more evident after deposition of Pass 2. The average Pass 2 bead height increased from 1.361 mm at 12H to 1.525 mm at 6H, corresponding to an approximately 12.0% increase. In comparison, the bead width changed only from 6.434 mm at 12H to 6.278 mm at 6H, representing a reduction of approximately 2.4%. These results demonstrate that the circumferential position influenced bead height more strongly than bead width.

Visual inspection of the reconstructed bead profiles further showed that local geometrical irregularities were concentrated at the 6H position. Such features were not clearly observed at 12H under the corresponding welding conditions. At the lower position, localized reinforcement, asymmetric toe transition, and accumulation of deposited metal were more apparent. This indicates that the effect of gravity is not limited to a simple translation of the molten pool but also influences the local distribution of molten metal and the final solidification geometry.

### 3.2. Quantitative Analysis of Bead Geometry

To quantitatively evaluate the positional effect, bead geometry was characterized using three-dimensional surface profiles. The first and last 10% of each measured profile were excluded from the analysis to minimize the influence of transient welding and measurement effects near the boundaries. The 10% exclusion was adopted as a consistent preprocessing criterion rather than as a statistically optimized or universal threshold, and the same criterion was applied to all experimental conditions and both circumferential positions to avoid condition-dependent selection of the analyzed region. The remaining central 80% of the profile was used to calculate the mean bead width and height as well as their spatial standard deviations.

The layer-by-layer comparison revealed a systematic evolution of bead geometry from Pass 1 to Pass 2. The mean values and standard deviations for the reference condition are compared in Figure 7 and Figure 8 for Pass 1 and Pass 2, respectively. Because Pass 1 was deposited under identical root-pass conditions, it provided a common baseline for evaluating the subsequent response of the weld to Pass 2 process parameters. Despite the identical root-pass parameters, a clear positional difference was present: the 6H position generally produced a higher and slightly narrower root bead than the 12H position.

After Pass 2 deposition, the influence of both welding position and process parameters became more pronounced. For the C1–C6 conditions, the average Pass 2 height was 1.361 ± 0.215 mm at 12H and 1.525 ± 0.208 mm at 6H. The corresponding widths were 6.434 ± 0.802 and 6.278 ± 0.78 mm. Therefore, the most consistent positional change was an increase in bead height at 6H rather than a large variation in bead width.

The response of bead geometry to the Pass 2 process parameters was also examined relative to the reference condition (N). It used a primary current of 180 A, background current of 105 A, travel speed of 2.54 mm/s, and wire speed of 9.31 mm/s. The individual C cases were designed by varying the primary current, wire feed speed, or travel speed while maintaining the remaining parameters.

Among these variables, the effect of wire feed speed on bead width was particularly consistent. In C3, where the wire speed was increased from 9.31 to 10.16 mm/s, the Pass 2 bead width increased by approximately 25.6% at 12H and 11.4% at 6H relative to reference condition. Conversely, in C4, where the wire speed was reduced from 9.31 to 8.47 mm/s, the bead width decreased at both positions. This opposing response indicates a direct relationship between filler metal supply and lateral bead development.

The influence of travel speed was most evident in C6. Increasing the travel speed from 2.54 to 2.96 mm/s reduced both the nominal heat input and filler metal deposition per unit length. As a result, the Pass 2 bead height decreased markedly relative to reference condition, by approximately 43.7% at 12H and 28.4% at 6H. This was the largest reduction among the investigated conditions and demonstrates that excessive travel speed strongly suppresses bead buildup.

Changes in primary current produced a different geometrical response. Reducing or increasing the primary current did not result in a monotonic change in bead height. For example, C2, which employed a higher primary current than reference condition, exhibited a lower bead height while its width increased. This suggests that increased arc energy may promote lateral spreading and remelting rather than simply increasing reinforcement.

Visual inspection of the reconstructed 3D profiles additionally revealed local differences in bead shape between the two positions. At 12H, the deposited bead generally exhibited a more laterally distributed profile, whereas at 6H localized vertical buildup and asymmetric transitions between the bead and groove surface were more apparent. These observations were consistent with the quantitative differences in bead height and width and indicate that welding position affected not only the average bead dimensions but also the local bead morphology.

### 3.3. Influence of Welding Parameters and Position on Weld-Bead Geometry

The influence of welding position was further investigated by comparing the modified welding conditions with the reference condition at the 12H and 6H positions. The C cases were grouped according to the independently varied parameter: primary current (C1 and C2), wire feed speed (C3 and C4), and travel speed (C5 and C6). This paired comparison was used to examine whether increasing or decreasing each parameter from the reference condition produced consistent changes in bead geometry and whether these responses depended on circumferential position.

For C1 and C2, the primary current was varied from the reference value of 180 A to 170 and 190 A, respectively, while the other welding parameters were maintained as constant. The bead height and width with respect to the different welding current is shown in Figure 9. At the low-current condition (C1), the bead height decreased relative to reference condition at both positions, while the width increased at 12H and remained nearly unchanged at 6H. Increasing the current to 190 A (C2) resulted in a further decrease in bead height, particularly at 12H, accompanied by an increase in bead width. Therefore, the variation from C1 through reference condition to C2 did not produce a monotonic increase in bead height with increasing current. Instead, the higher current condition showed a greater tendency toward a lower and wider bead. This behavior suggests that primary current affected the balance between vertical reinforcement and lateral molten-pool spreading rather than simply controlling the overall bead size. At lower current, reduced arc energy limited melting and bead buildup, whereas at higher current, increased arc energy likely enhanced melting, pool fluidity, and lateral spreading. This effect was more pronounced at 12H, while the height reduction was smaller at 6H. The difference indicates that the geometrical response to current variation was modified by welding position, with gravitationally influenced molten-metal retention at 6H contributing to the maintenance of reinforcement.

The effect of filler metal supply was evaluated by varying the wire feed speed from the reference condition value of 9.31 to 10.16 mm/s in C3 and 8.47 in C4. The bead height and width with respect to the different wire feed speeds are shown in Figure 10. A clear and consistent relationship was observed between wire feed speed and bead width. Increasing the wire feed speed in C3 increased the bead width relative to reference condition at both positions, whereas decreasing it in C4 reduced the width. These opposite responses confirm that the amount of filler metal supplied to the molten pool was a major factor controlling lateral bead development. In contrast, the bead height response showed a stronger dependence on welding position. At 12H, both the increased and decreased wire feed conditions resulted in lower heights than reference condition, whereas at 6H the bead height was maintained or increased. In particular, the additional filler metal supplied in C3 contributed predominantly to lateral spreading at 12H but resulted in both bead widening and increased reinforcement at 6H. These results suggest that wire feed speed primarily determines the amount of deposited metal, while circumferential position influences how this metal is distributed between lateral spreading and vertical reinforcement.

For C5 and C6, the travel speed decreased from the reference condition value of 2.54 to 2.12 and increased to 2.96 mm/s, respectively. The bead height and width with respect to the different travel speeds are shown in Figure 11. Decreasing the travel speed in C5 increased both the nominal heat input and filler metal deposition per unit length. However, this did not result in a corresponding increase in bead dimensions. Instead, the bead height and width decreased at 12H, whereas the height at 6H remained close to that of reference condition. In contrast, increasing the travel speed in C6 reduced both heat input and filler deposition per unit length and produced the most pronounced reduction in bead height among the investigated conditions. The travel speed results demonstrate that bead formation was governed not only by nominal heat input but also by molten-pool residence time, filler deposition, and gravitational redistribution. At the lower travel speed, the longer molten-pool lifetime provided more time for remelting and redistribution, which may explain why the increased heat input did not directly translate into greater reinforcement. At the higher travel speed, reduced heat input and filler deposition strongly suppressed bead buildup. Nevertheless, the smaller height reduction at 6H indicates that gravitationally influenced accumulation partially maintained the reinforcement even under reduced deposition conditions.

Overall, the paired comparisons of C1–C2, C3–C4, and C5–C6 demonstrate that the effect of each welding parameter was dependent on circumferential position. Primary current mainly influenced the balance between melting and lateral spreading, wire feed speed showed the clearest relationship with bead width, and travel speed strongly affected bead buildup through simultaneous changes in heat input, filler deposition, and molten-pool residence time. The generally greater bead height at 6H indicates that gravitational orientation favored local accumulation of molten metal, whereas the 12H position promoted greater redistribution and lateral spreading. Therefore, the final bead geometry in orbital GTAW was determined by the interaction between welding parameters and gravity-dependent molten-pool behavior rather than by individual process parameters alone.

### 3.4. Mechanism of Gravity-Induced Bead Distortion

The experimental observations suggest that the geometrical defects observed at the lower welding position originate from the interaction between gravity-driven molten-metal flow and the cumulative nature of multi-pass deposition.

During orbital welding, the orientation of the molten pool continuously changes relative to gravity. Even when the welding current, wire feed, and travel speed remain constant, the balance among gravity, surface tension, arc pressure, viscous resistance, and solidification varies with circumferential position. Therefore, an initially uniform welding condition does not necessarily produce a uniform bead geometry around the entire circumference.

At 6H, gravity promotes downward accumulation of molten metal in the lower portion of the weld pool. If the rate of filler metal addition exceeds the rate at which the molten metal can spread laterally before solidification, local reinforcement increases. This produces a higher bead profile and may simultaneously shift the toe position or generate an asymmetric transition between the deposited bead and the surrounding surface.

The effect becomes particularly important in multi-pass welding because the geometry produced by one pass defines the boundary condition for the next pass. A small positional distortion in the root or preceding layer modifies the local torch to workpiece distance, groove volume, wetting condition, and direction of subsequent molten-metal flow. The next deposited layer therefore does not form on an ideal, uniform substrate but on an already position-dependent surface.

The Pass 1 and Pass 2 results support this interpretation. A positional height difference was already present in Pass 1 under identical welding conditions, and the difference became larger after Pass 2. The increase from approximately 9.6% in Pass 1 to approximately 12.0% in Pass 2 suggests that the positional effect can propagate through successive layers rather than being completely corrected by the subsequent pass.

Changes in welding parameters may further amplify this mechanism. Increased wire feed increases the quantity of molten filler metal that must be accommodated by the pool, whereas reduced wire feed limits available deposition volume. Changes in travel speed simultaneously alter heat input, filler metal deposition per unit length, and the residence time of the molten pool. Therefore, the same parameter change may produce different geometrical responses at 12H and 6H.

This interaction provides a plausible explanation for the occurrence of geometrical defects preferentially at 6H. Local excessive reinforcement or asymmetric bead accumulation can create unfavorable inter-pass overlap or toe geometry and thereby modify the geometrical boundary conditions for subsequent deposition. However, the present 3D surface measurements characterize only external bead morphology and do not directly provide information regarding internal defects, penetration, or fusion quality. Therefore, no direct causal relationship between the observed surface geometry and internal weld defects is claimed in the present study. Future work will combine three-dimensional bead-profile measurements with radiographic examination and cross-sectional analysis to determine whether specific geometrical indicators, such as excessive reinforcement, toe displacement, or abnormal overlap, are associated with internal weld quality.

Overall, the results demonstrate that gravitational effects during orbital GTAW should be considered as a cumulative, position-dependent process rather than a single pass geometrical effect. The 6H position is particularly sensitive because gravity modifies molten-metal redistribution and can amplify local geometrical deviations generated during preceding passes.

## 4. Selection of Position-Dependent Welding Parameters for Bead Geometry Matching

### 4.1. Selection of Position-Dependent Welding Parameters

The results presented in Section 3 demonstrated that identical welding parameters at the 12H and 6H positions did not produce identical bead geometries. The positional difference was particularly evident in bead height, while the variation in bead width was comparatively smaller. In addition, the response of bead geometry to changes in primary current, wire feed speed, and travel speed depended on circumferential position. These results indicate that uniform bead geometry around the pipe circumference cannot be achieved solely by maintaining constant welding parameters; instead, position-dependent adjustment of the welding conditions is required.

Among the investigated parameters, primary current was selected as the principal variable for position-dependent compensation. The primary-current experiments consisted of C1 (170 A), N (180 A), and C2 (190 A), while the background current, travel speed, and wire feed speed were maintained at 105 A, 2.54 mm/s, and 9.31 mm/s, respectively. Because only the primary current was varied in these cases, the influence of current on bead height and width could be evaluated without simultaneously changing filler metal deposition or travel speed. In comparison, variation in wire feed speed predominantly affected bead width, whereas travel speed simultaneously altered heat input, filler metal deposition per unit length, and molten-pool residence time. Therefore, primary current was considered the most suitable variable for first-stage compensation of the positional bead geometry difference.

To determine a pair of welding conditions capable of producing similar bead geometries at 12H and 6H, the measured Pass 2 bead height and width obtained under C1, N, and C2 were considered as functions of primary current for each welding position. Rather than selecting the geometry of one reference condition as the target, the objective was to identify two position-dependent primary-current values at which the predicted bead height and width converged simultaneously. Based on interpolation within the experimentally investigated current range, the matching condition was estimated to be approximately 173 A at 12H and 175.5 A at 6H. These values are referred to as predicted position-dependent matching conditions rather than optimum welding parameters because they were derived by interpolation of the experimentally observed geometrical responses and were not determined or validated using a formal optimization methodology.

Under the proposed conditions, the background current, travel speed, and wire feed speed were maintained at 105 A, 2.54 mm/s, and 9.31 mm/s, respectively. The predicted Pass 2 bead geometry was approximately 1.62 mm in bead height and 6.57 mm in bead width at both welding positions as shown in Table 3. Thus, a relatively small increase in primary current at 6H compared with 12H was predicted to compensate for the position-dependent molten-pool behavior and reduce the difference in final bead geometry.

The proposed values should be interpreted as predicted matching conditions rather than experimentally confirmed optimum parameters. However, the selected values lie within the experimentally investigated range of 170–190 A and therefore represent interpolation rather than extrapolation. This reduces the uncertainty associated with the prediction and provides a reasonable basis for subsequent experimental verification. The result also demonstrates that achieving circumferentially uniform bead geometry does not necessarily require identical welding parameters at all positions. Instead, the welding parameters can be adjusted according to circumferential position so that different process conditions produce similar final bead dimensions.

### 4.2. Engineering Implications

The proposed position-dependent parameter-selection strategy has direct implications for automated orbital GTAW. During circumferential welding, the orientation of the molten-pool relative to gravity changes continuously, causing the same nominal welding condition to produce different molten pool flow and solidification behavior at different positions. As discussed in Section 3, these differences can persist between successive passes and contribute to position-dependent bead geometry. Therefore, maintaining constant process parameters around the entire circumference does not necessarily ensure geometrical uniformity.

The present results suggest that this positional variation can be compensated by applying position-dependent welding parameters. In particular, the predicted matching condition requires only a relatively small difference in primary current between the 12H and 6H positions while the remaining parameters are maintained constant. The predicted difference in primary current between the two positions is approximately 2.5 A and is therefore relatively small compared with the overall welding current level. Its practical implementation must consequently be evaluated in relation to the current control resolution and stability of the welding power source, bead geometry measurement uncertainty, and normal run-to-run process variability. The present interpolation indicates that the predicted geometry matching conditions at the two positions do not necessarily occur at identical current values; however, it does not demonstrate that a 2.5 A adjustment will necessarily produce a statistically or practically significant improvement under industrial welding conditions. Experimental validation using the proposed current values, together with an uncertainty analysis of current control and bead geometry measurement, is therefore required before the engineering significance of this compensation level can be established. This approach is attractive for automated orbital welding because primary current can be continuously controlled as a function of torch position without changing the mechanical configuration of the welding system.

Rather than implementing abrupt parameter changes only at discrete positions such as 12H and 6H, the proposed concept can be extended to a continuous circumferential current profile. The welding current could be gradually adjusted according to the angular position of the torch so that gravity-induced variations in molten-metal distribution are compensated throughout the entire circumference. Such position-dependent control would shift the process objective from maintaining identical input parameters to maintaining consistent output geometry. External bead geometry, however, represents only one aspect of overall weld quality. Comparable bead height and width do not necessarily indicate equivalent penetration, fusion quality, internal defect population, microstructure, or mechanical strength. Nevertheless, geometrical characteristics such as reinforcement, bead width, toe position, and inter-pass overlap define the local boundary conditions for subsequent deposition and may influence remelting and fusion behavior during successive passes. Therefore, the position-dependent parameter-selection approach proposed in this study should be interpreted as a strategy for controlling external bead geometry rather than as direct evidence of improved overall weld quality. Further validation combining three-dimensional surface measurements with cross-sectional metallography, nondestructive examination, and mechanical testing is required to determine how the observed geometrical differences and their compensation affect penetration, fusion, internal weld integrity, and mechanical performance.

It should be noted that the present investigation was limited to the 12H and 6H regions, representing the upper/overhead and lower/bottom portions of the pipe, respectively. The 3H and 9H side positions were not independently characterized. Because the orientation of gravity relative to the molten-pool changes continuously around the pipe circumference, molten-metal redistribution and the resulting bead geometry at the side positions may differ from those observed at 12H and 6H. Therefore, the proposed position-dependent conditions should be interpreted as predicted matching conditions for the two experimentally investigated regions rather than as experimentally validated conditions for the entire circumference. Extension of the present measurements to intermediate circumferential positions will be required to establish a continuous position-dependent parameter profile around the pipe. The present parameters are limited to bead height and width and are based on the experimentally observed response within the investigated parameter range. Therefore, the predicted conditions should not yet be interpreted as guaranteeing equivalent penetration, fusion behavior, or internal weld quality. Nevertheless, the proposed position-dependent compensation strategy provides a practical framework for reducing circumferential bead geometry variation and forms a basis for future closed-loop or position adaptive control of multi-pass orbital GTAW.

## 5. Conclusions

This study investigated the effect of circumferential welding position and process parameters on bead geometry during multi-pass orbital GTAW. Three-dimensional surface profiles were used to quantitatively evaluate the bead height and width at the 12H and 6H positions. To minimize transient edge effects, the first and last 10% of the measured region were excluded, and the central 80% was used for quantitative analysis. Based on the experimental results and the position-dependent parameter analysis, the following conclusions were obtained:

1. Differences in bead geometry were observed between the 12H and 6H positions even under identical welding conditions. For Pass 1, which was deposited using the same root-pass conditions for all cases, the average bead height at 6H was approximately 9.6% greater than that at 12H, whereas the bead width was approximately 3.3% smaller. After Pass 2 deposition, the difference in bead height increased to approximately 12.0%, while the difference in bead width remained relatively small at approximately 2.4%. Within the present experimental dataset, the positional difference was more apparent in bead height than in bead width. In particular, the relatively small differences in bead width should be interpreted cautiously considering the observed within-weld spatial variability.
2. The effect of the welding parameters on bead geometry depended on circumferential position. Primary-current variation affected the balance between vertical bead buildup and lateral molten-pool spreading, and increasing the primary current did not result in a monotonic increase in bead height. Wire feed speed showed the clearest relationship with bead width: increasing the wire feed speed increased the bead width, whereas decreasing it reduced the width at both positions. Travel speed strongly affected bead buildup because it simultaneously altered heat input, filler metal deposition per unit length, and molten-pool residence time. In particular, the high travel speed condition produced the largest reduction in Pass 2 bead height.
3. The different responses at 12H and 6H indicate an interaction between welding parameters and gravity-dependent molten-pool behavior. The generally greater bead height observed at 6H suggests that the gravitational orientation favored local accumulation of molten metal, whereas the 12H position showed a greater tendency toward redistribution and lateral spreading. The persistence of the positional difference from Pass 1 to Pass 2 further indicates that the effect of welding position was not eliminated by subsequent deposition and could influence the geometrical development of multi-pass welds.
4. Position-dependent welding parameters were proposed to minimize the bead geometry difference between 12H and 6H. Based on interpolation of the primary-current experiments (C1–N–C2), a primary current of approximately 173 A at 12H and 175.5 A at 6H, with the background current, travel speed, and wire feed speed maintained at 105 A, 2.54 mm/s, and 9.31 mm/s, respectively, was predicted to produce similar Pass 2 geometries. The corresponding bead height and width were predicted to converge to approximately 1.62 mm and 6.57 mm, respectively. This result suggests that circumferential bead uniformity may be improved by adjusting the welding parameters according to position rather than maintaining identical parameters throughout the entire circumference.

It should be emphasized that each welding condition was experimentally tested once, while the reported mean values and standard deviations were calculated from spatially resolved profiles within each weld. Therefore, the standard deviations represent within-weld spatial variability rather than variability among independent experimental repetitions. Accordingly, the differences and parameter–response relationships reported in this study should be interpreted as descriptive experimental trends and not as statistically significant differences between independently replicated welding conditions. Additional welding repetitions and formal statistical analysis will be required to establish the statistical significance and reproducibility of these observed trends.

## Figures and Tables

**Figure 1:**
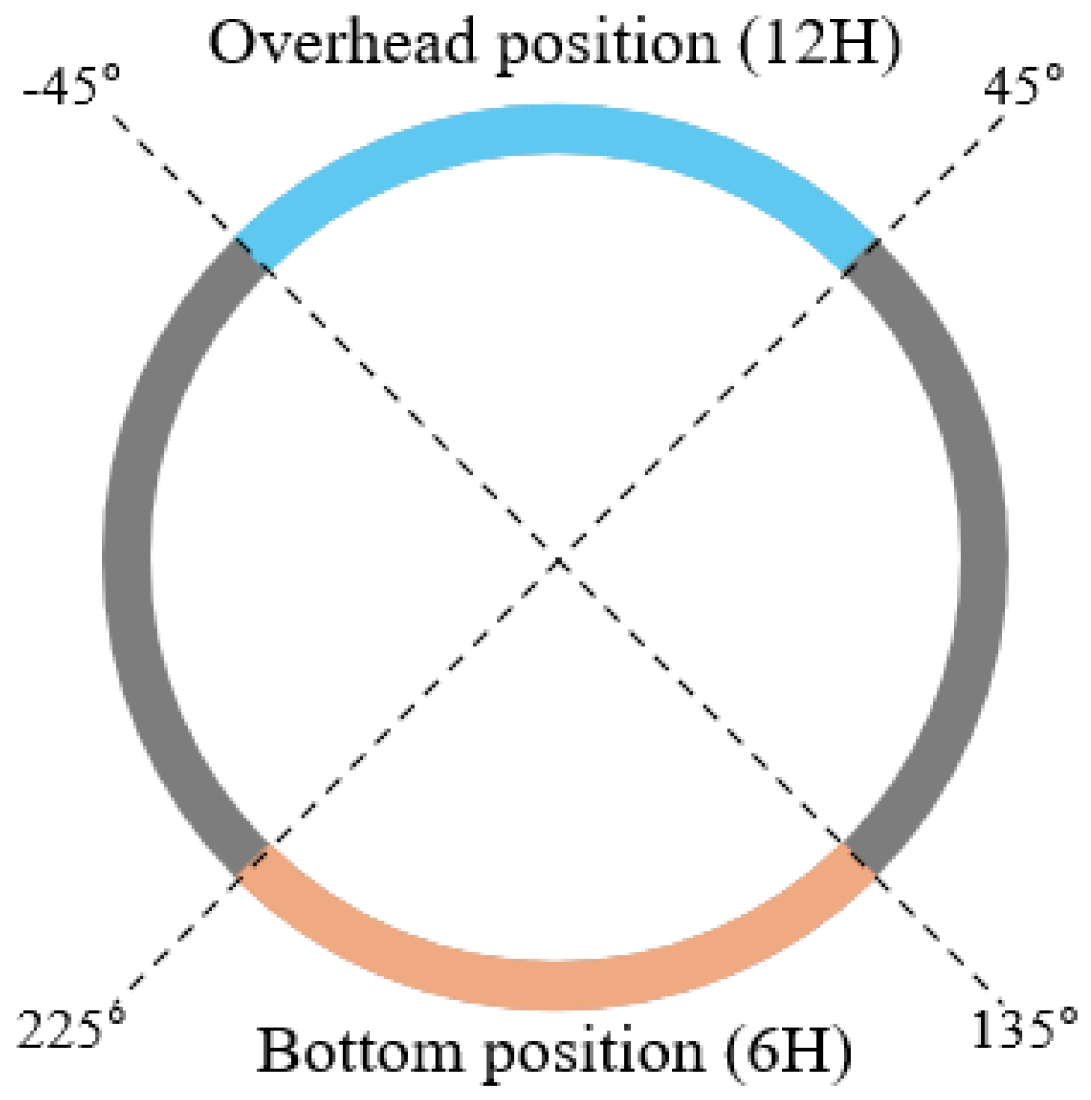
Definition of the circumferential welding positions used for bead geometry analysis: 12H (−45° to 45°) and 6H (135° to 225°), with 0° defined at the top of the pipe.

**Figure 2:**
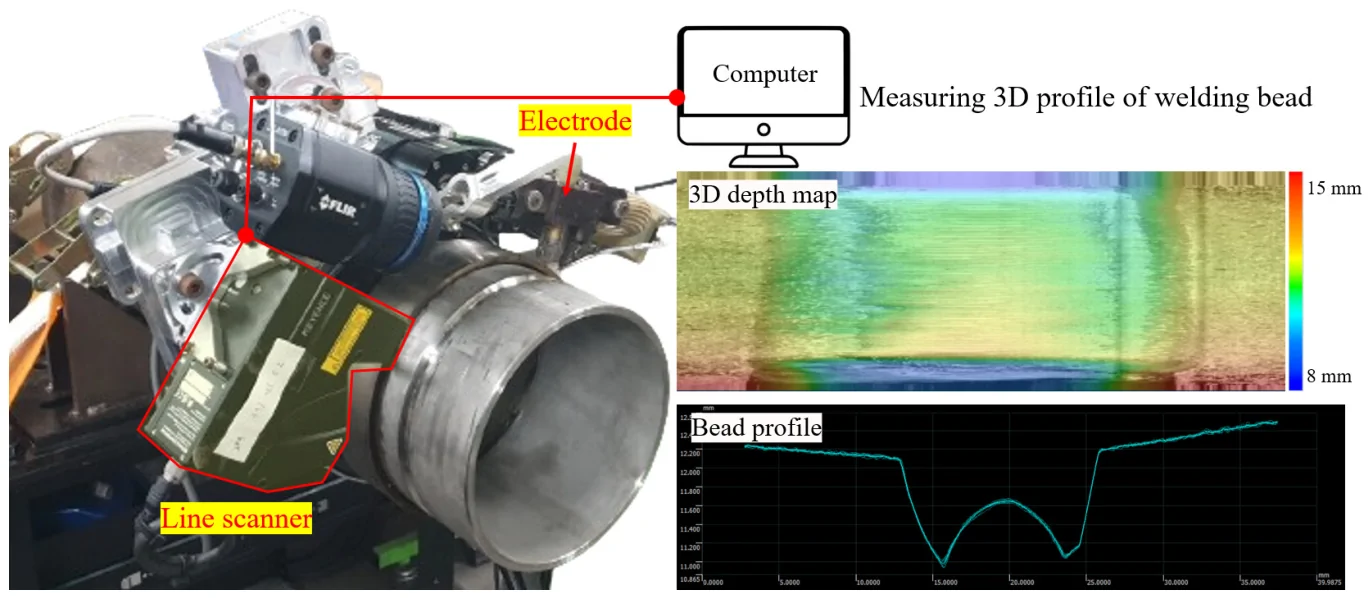
Experimental configuration of the orbital GTAW system and laser line scanner for three-dimensional weld-bead measurement, with representative 3D depth map and extracted cross-sectional bead profile.

**Figure 3:**
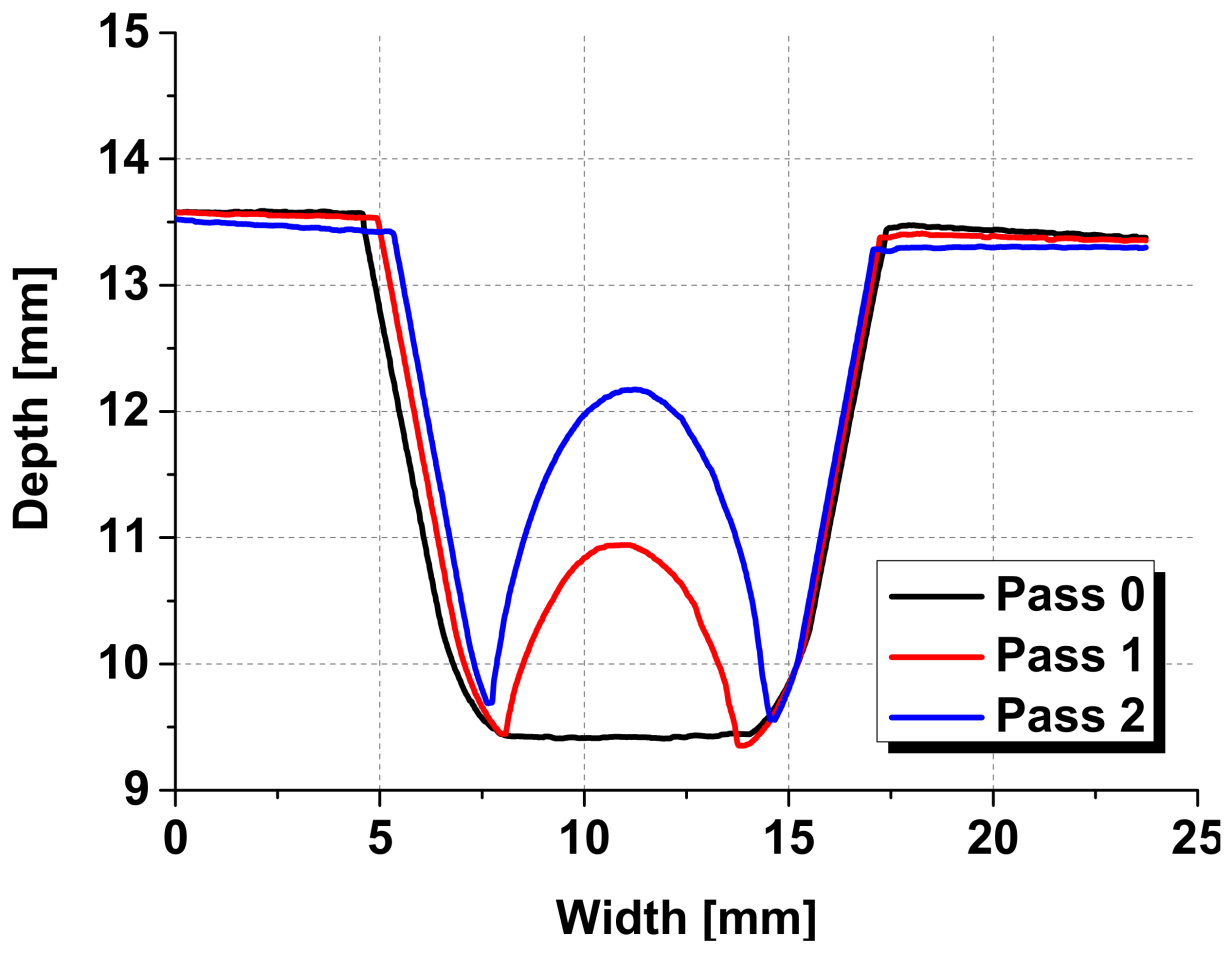
Representative superposition of the measured Pass 0, Pass 1, and Pass 2 profiles, illustrating the potential uncertainty in determining individual bead dimensions by direct profile comparison due to welding-induced deformation in the measurement reference.

**Figure 4:**
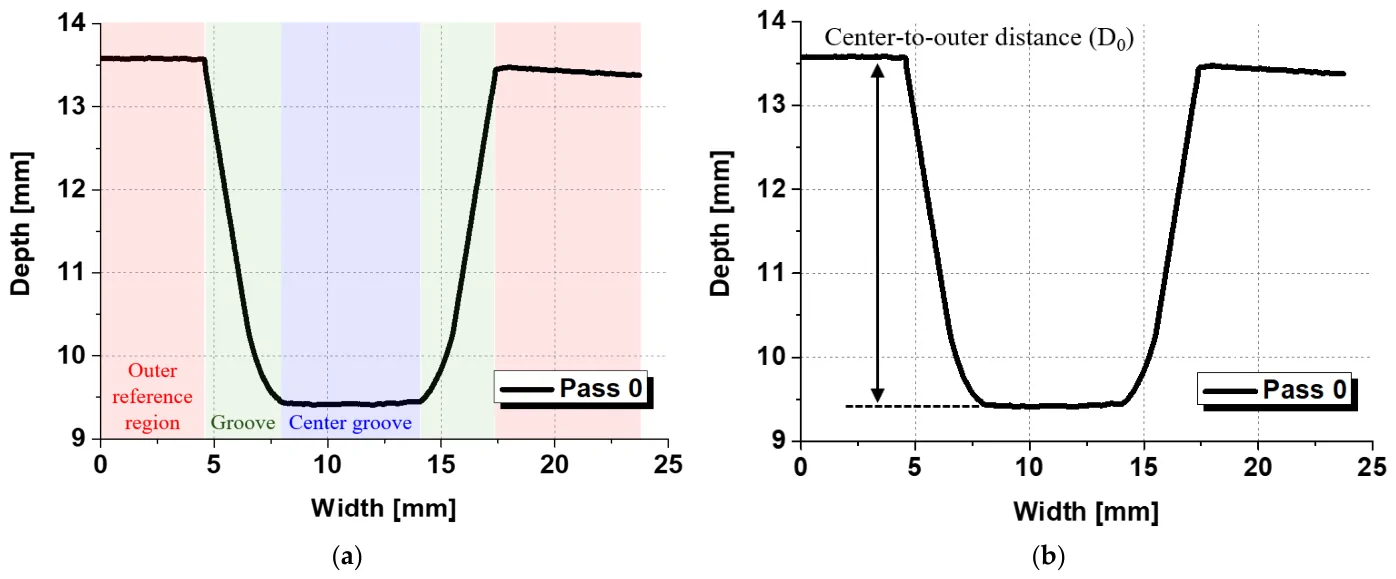
Overall reference region determination based on Pass 0; (**a**) reference region of bead geometry; (**b**) center-to-outer distance of pass 0.

**Figure 5:**
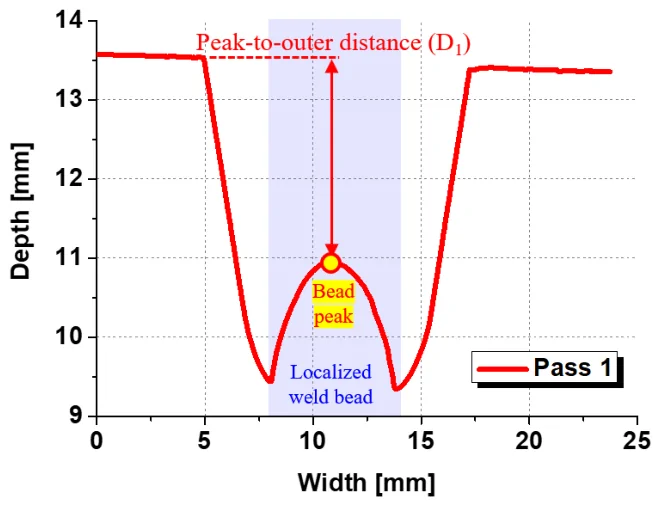
Measuring bead peak and peak-to-outer distance at the weld bead.

**Figure 6:**
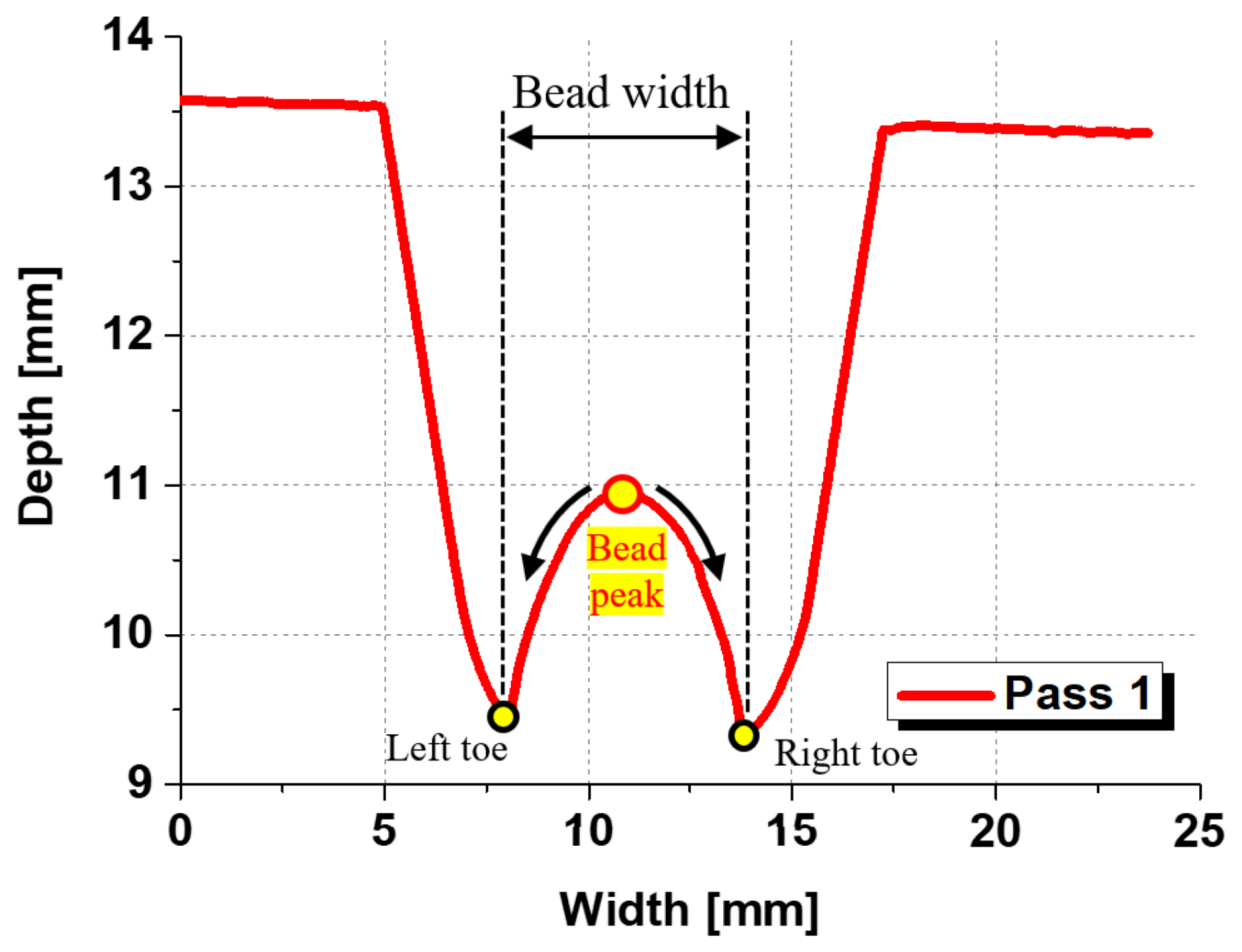
Measuring bead width based on left and right toe.

**Figure 7:**
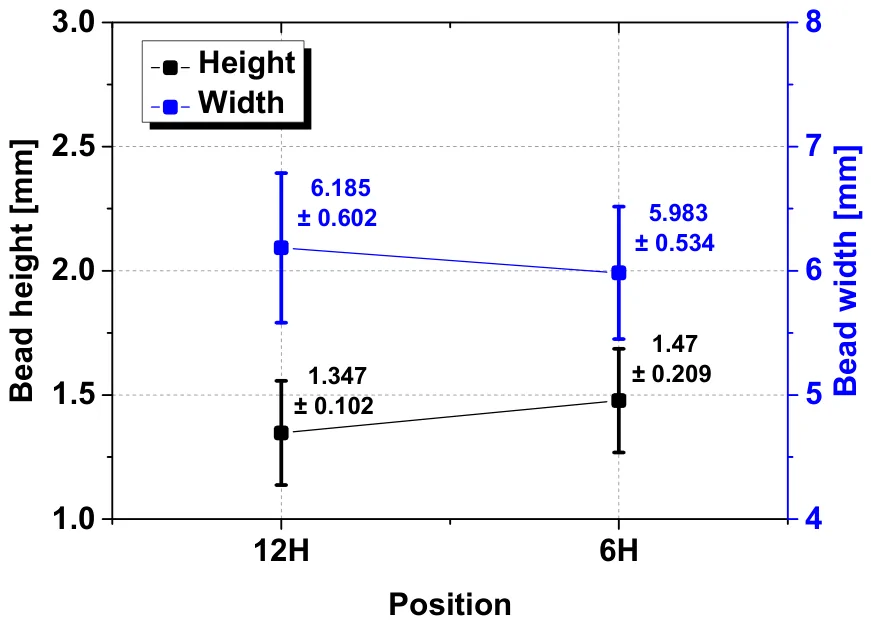
Comparison of Pass 1 bead height and width for reference conditions at 12H and 6H. Values are presented as the mean ± one spatial standard deviation (SD) calculated from the spatial profile datasets within the analyzed weld region.

**Figure 8:**
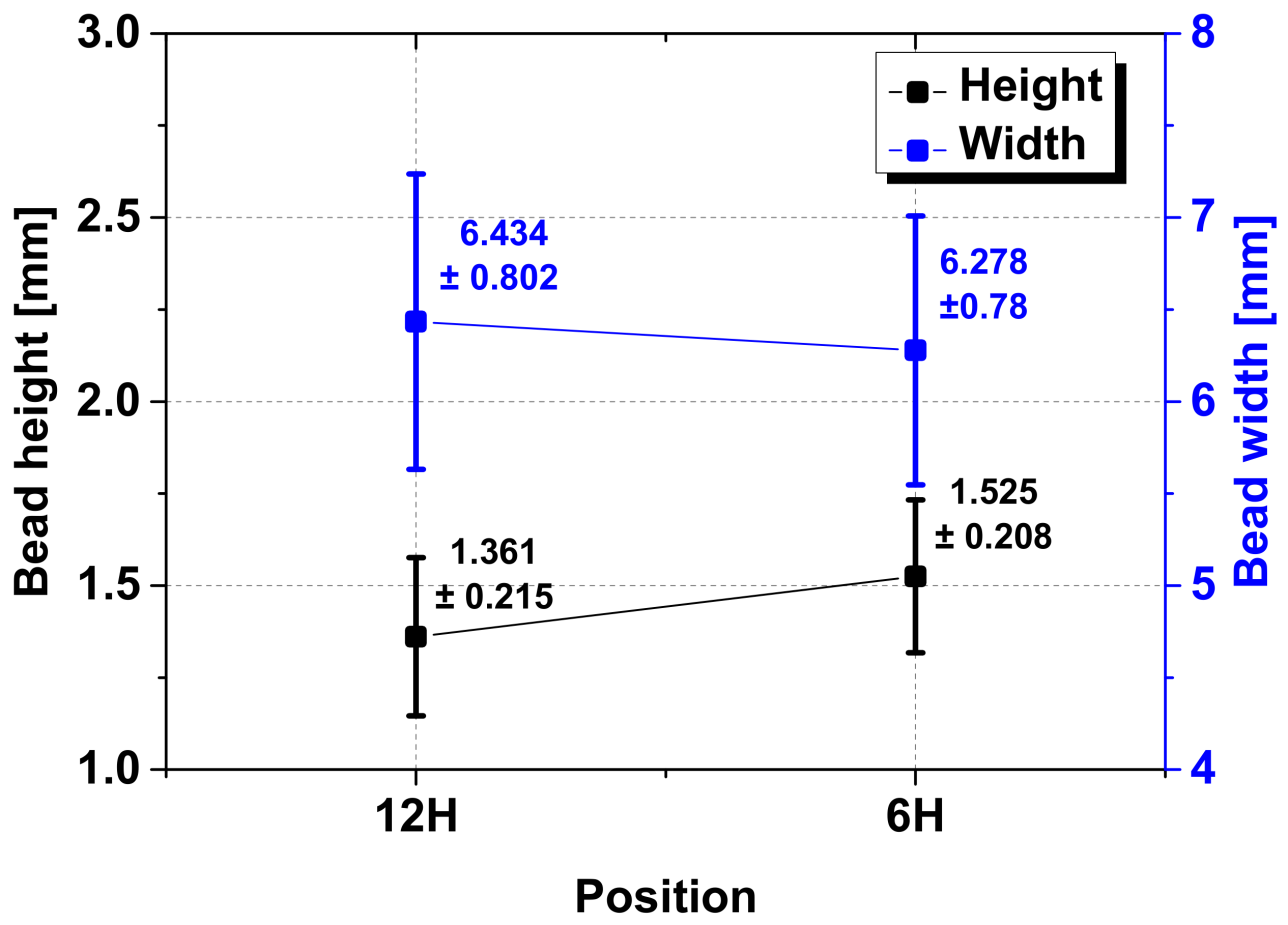
Comparison of Pass 2 bead height and width for reference conditions at 12H and 6H. Values are presented as the mean ± one spatial standard deviation (SD) calculated from the spatial profile datasets within the analyzed weld region.

**Figure 9:**
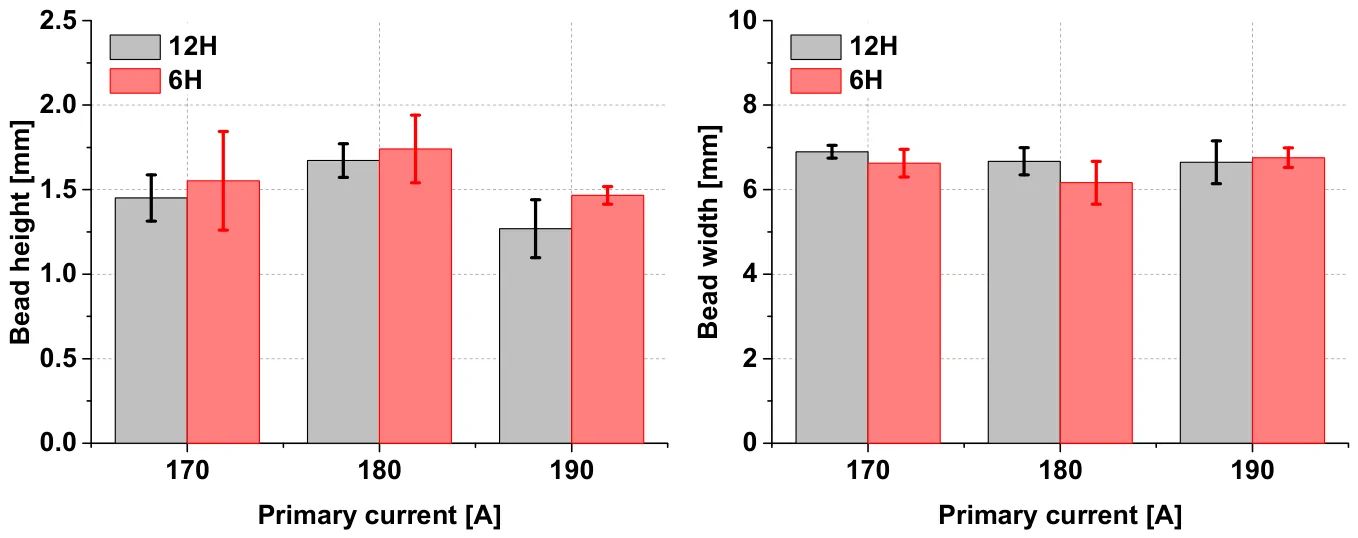
Pass 2 bead width and height with respect to primary welding current at the 12H and 6H positions.

**Figure 10:**
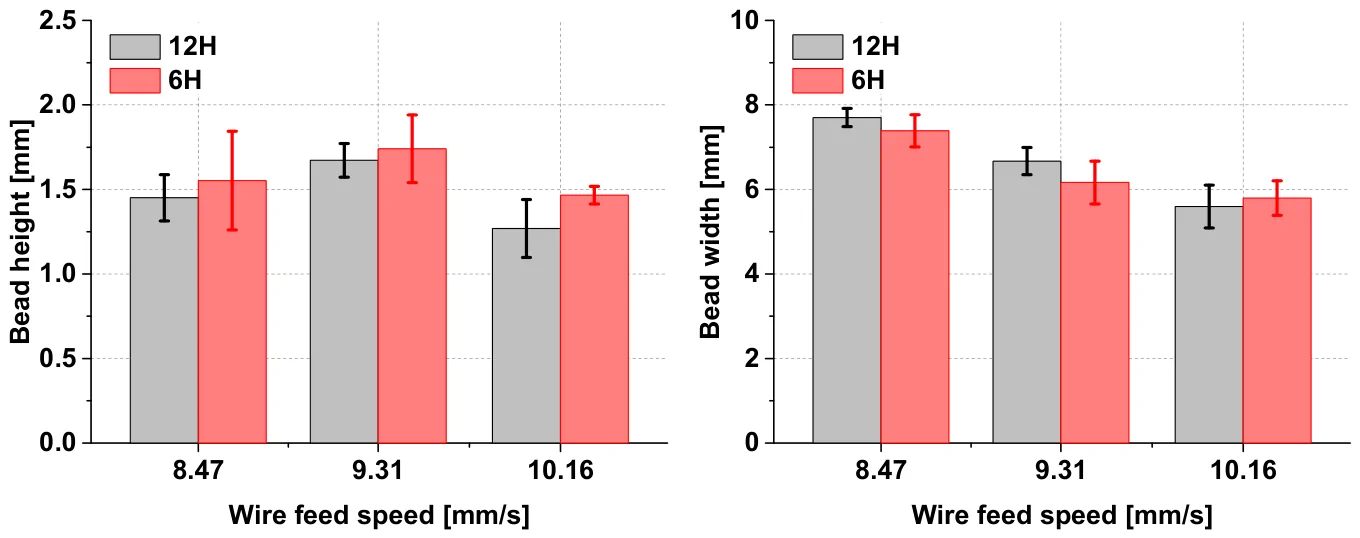
Pass 2 bead width and height with respect to wire feed speed at the 12H and 6H positions.

**Figure 11:**
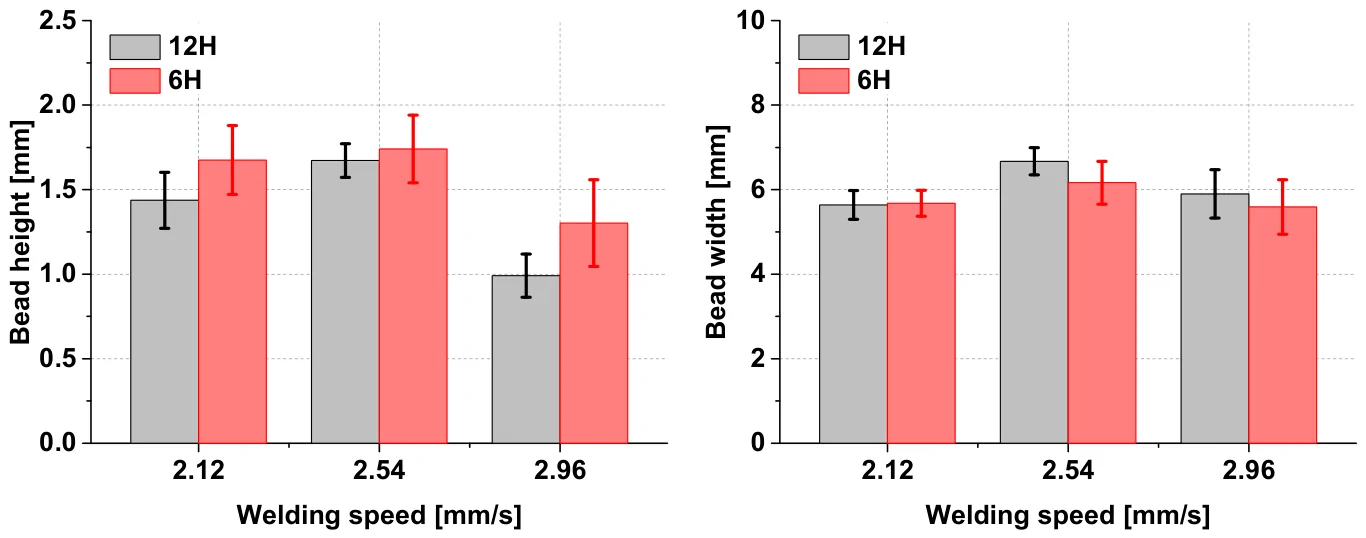
Pass 2 bead width and height with respect to travel speed at the 12H and 6H positions.

**Table 1:** Chemical composition of the SUS304 pipe used in the present study (wt%) [36].

	C	Mo	Si	Mn	P	S	Ni	Cr	Fe
SUS304	0.04	0	0.53	0.91	0.037	0.001	8.08	18.2	bal.

**Table 2:** Additional parameter sets prepared for parameter analysis.

Test Case	Primary Welding Current [Amp]	Background Welding Current [Amp]	Travel Speed [mm/s]	Wire Feed Speed [mm/s]	Welding Time [s]
C1	170	105	2.54	9.31	66
C2	190	105	2.54	9.31	66
C3	180	105	2.54	10.16	66
C4	180	105	2.54	8.47	66
C5	180	105	2.12	9.31	79
C6	180	105	2.96	9.31	56

**Table 3:** Predicted position-dependent welding parameters for bead geometry matching.

Welding Parameter	12H Proposed Condition	6H Proposed Condition
Primary current	173 Amp	175.5 Amp
Background current	105 Amp	105 Amp
Travel speed	2.54 mm/s	2.54 mm/s
Wire feed speed	9.31 mm/s	9.31 mm/s
Predicted bead height	1.62 mm	
Predicted bead width	6.57 mm	

## Data Availability

The original contributions presented in this study are included in the article. Further inquiries can be directed to the corresponding author.

## References

[1] Minh P.S., Nguyen V.-T., Do T.T., Uyen T.M.T., Toan H.D.S., Linh H.T.T., Nguyen V.T.T. (2024). Parameter optimization in orbital TIG welding of SUS 304 stainless steel pipe. Metals.

[2] Baskoro A.S., Kiswanto G., Widyianto A. (2020). Optimization of PC-GTAW orbital welding parameters of AISI 304L stainless steel pipe using ANOVA and Taguchi method. IOP Conf. Ser. Mater. Sci. Eng..

[3] Figueirôa D.W., Pigozzo I.O., Silva R.H.G., Santos T.F.A., Filho S.L.U. (2017). Influence of welding position and parameters in orbital TIG welding applied to low-carbon steel pipes. Weld. Int..

[4] Na S.J., Lho T.J. (1996). A study on parameter optimization for circumferential gas tungsten arc welding of small pipes considering backing gas pressure, Part 1: Analysis of weld pool surface profile. Proc. Inst. Mech. Eng. Part B J. Eng. Manuf..

[5] Li X.R., Shao Z., Zhang Y., Kvidahl M. (2013). Monitoring and control of penetration in GTAW and pipe welding. Weld. J..

[6] Kim W.H., Na S.J. (1998). Heat and fluid flow in pulsed current GTA weld pool. Int. J. Heat Mass Transf..

[7] Huang X., Kang N., Wang Q., El Mansori M., Guittonneau F. (2025). Wear of directed energy deposited H13 steel as a function of its graded microstructure. Tribol. Int..

[8] Tran N.-T., Nguyen V.-T., Do T.T., Hoang V.H., Ngoc Huy D., Tan Phat H., Nguyen V.T.T. (2025). Parameter Optimization of Orbital TIG Welding on Stainless Steel Pipe. Appl. Sci..

[9] Kang N., Mahank T.A., Kulkarni A.K., Singh J. (2003). Effects of gravitational orientation on surface deformation and weld pool geometry during gas tungsten arc welding. Mater. Manuf. Process..

[10] Kang N., Cho K., Park Y.D., Singh J., Kulkarni A.K. (2008). Behavior of weld pool shape and weld surface deformation as a function of spot-GTA welding position for 304 stainless steel. J. Weld. Join..

[11] Kang N., Singh J., Kulkarni A.K. (2004). Gravitational effects on the weld pool shape and microstructural evolution during gas tungsten arc and laser beam welding of 304 stainless steel and Al–4 wt% Cu alloy. Ann. N. Y. Acad. Sci..

[12] Traidia A., Roger F., Schroeder J., Guyot E., Marlaud T. (2013). On the effects of gravity and sulfur content on the weld shape in horizontal narrow gap GTAW of stainless steels. J. Mater. Process. Technol..

[13] Fan H.G., Tsai H.L., Na S.J. (2001). Heat transfer and fluid flow in a partially or fully penetrated weld pool in gas tungsten arc welding. Int. J. Heat Mass Transf..

[14] Kim W.H., Fan H.G., Na S.J. (1997). Effect of various driving forces on heat and mass transfer in arc welding. Numer. Heat Transf. Part A Appl..

[15] Tsai M.C., Kou S. (1990). Electromagnetic-force-induced convection in weld pools with a free surface. Weld. J..

[16] Kim S.D., Na S.J. (1992). Effect of weld pool deformation on weld penetration in stationary gas tungsten arc welding. Weld. J..

[17] Choo R.T.C., Szekely J., David S.A. (1992). On the calculation of the free surface temperature of gas-tungsten-arc weld pools from first principles: Part II—Modeling the weld pool and comparison with experiments. Metall. Trans. B.

[18] Zacharia T., David S.A., Vitek J.M., DebRoy T. (1989). Weld pool development during GTA and laser beam welding of Type 304 stainless steel, Part I: Theoretical analysis. Weld. J..

[19] Zacharia T., David S.A., Vitek J.M., DebRoy T. (1989). Weld pool development during GTA and laser beam welding of Type 304 stainless steel, Part II: Experimental correlation. Weld. J..

[20] Zhang Y.M., Cao Z.N., Kovacevic R. (1996). Numerical analysis of fully penetrated weld pools in gas tungsten arc welding. Proc. Inst. Mech. Eng. Part C J. Mech. Eng. Sci..

[21] Choo R.T.C., Szekely J., Westhoff R.C. (1990). Modeling of high-current arcs with emphasis on free-surface phenomena in the weld pool. Weld. J..

[22] Yildirim M.S., Ayhan E., Aydin K., Durgutlu A. (2025). The effect of welding parameters on weld bead geometry in gas tungsten arc welding applications by response surface methodology model. Proc. Inst. Mech. Eng. Part E J. Process Mech. Eng..

[23] Barath Kumar M.D., Arivazhagan N., Tofil S., Andersson J., Kozak J., Manikandan M. (2024). Influence of pulsed current GTAW-WAAM process parameters on the single layer bead geometry and multi bead multi-layer deposition of a nickel-based superalloy. Mater. Today Commun..

[24] Ungethüm T., Spaniol E., Hertel M., Füssel U. (2020). Analysis of metal transfer and weld geometry in hot-wire GTAW with indirect resistive heating. Weld. World.

[25] Geng H., Li J., Xiong J., Lin X., Zhang F. (2017). Optimization of wire feed for GTAW based additive manufacturing. J. Mater. Process. Technol..

[26] Zacharia T., Eraslan A.H., Aidun D.K. (1988). Modeling of non-autogenous welding. Weld. J..

[27] Wang W., Chen R., Zhang Y., Wang Y., Zhang K. (2026). Prediction of multi-layer multi-pass weld bead full-profile based on fusion of welding parameters and bead geometry constraints. J. Manuf. Process..

[28] Truong V.D., Yoon J. (2026). Deep learning prediction of weld bead geometry during multi-pass multi-layer welding process. Eng. Appl. Artif. Intell..

[29] Li Y., Li Y.F., Wang Q.L., Xu D., Tan M. (2010). Measurement and defect detection of the weld bead based on online vision inspection. IEEE Trans. Instrum. Meas..

[30] Huang W., Kovacevic R. (2011). laser-based vision system for weld quality inspection. Sensors.

[31] Xu Y., Yu H., Zhong J., Lin T., Chen S. (2012). Real-time seam tracking control technology during welding robot GTAW process based on passive vision sensor. J. Mater. Process. Technol..

[32] Kim G.-G., Kim D.-Y., Yu J. (2024). Vision-Based Acquisition Model for Molten Pool and Weld-Bead Profile in Gas Metal Arc Welding. Metals.

[33] Yang L., Wang B., Liu P., Jeon I., Chen Z., Li M., Xiong Y., Cheng J.C.P., Sohn H. (2025). Laser line scanner based real-time geometry monitoring using Encoder-Decoder network during Directed Energy deposition. Measurement.

[34] Zacharia T., Eraslan A.H., Aidun D.K. (1988). Modeling of autogenous welding. Weld. J..

[35] Nishiguchi K., Ohji T., Nakata N., Ishibashi K. (1984). Study on behavior of molten pool in arc welding: Mathematical modeling of a molten pool in arc welding of thin plate. Q. J. Jpn. Weld. Soc..

[36] Okayasu M., Tomida S. (2017). Phase transformation system of austenitic stainless steels obtained by permanent compressive strain. Mater. Sci. Eng. A.

